# Conservative Management of a COVID-19-positive Patient With Emphysematous Pyelonephritis: A Case Report

**DOI:** 10.7759/cureus.33315

**Published:** 2023-01-03

**Authors:** Abdulaziz K AlMulhim, Ahmed Alamri, Ali AlAbandi, Mohammed AlDahoos, Abdullah Alfulij

**Affiliations:** 1 Urology, King Fahad Specialist Hospital, Dammam, SAU

**Keywords:** emphysematous pyelonephritis, conservative, antibiotics, infection, uti

## Abstract

Herein, we present a case of emphysematous pyelonephritis with septic shock that was treated conservatively. A 44-year-old woman with diabetes mellitus presented to the emergency department with acute abdominal discomfort. Clinical examination revealed that the patient was conscious but vitally unstable. Therefore, the patient required inotropic support. A computed tomography scan revealed gas in the left kidney, suggestive of emphysematous pyelonephritis. Subsequently, the patient was treated conservatively and stabilized with broad-spectrum antibiotics, strict blood glucose management, and drainage.

## Introduction

The presence of gas in the kidneys as a result of gas-producing organisms is known as emphysematous pyelonephritis (EPN) [1,2]. The clinical presentation of EPN includes mild urinary symptoms of septic shock. Herein, we describe a case of EPN that was conservatively treated with drainage and antibiotics [1-3].

## Case presentation

A 44-year-old woman who had a history of diabetes mellitus and did not adhere to her oral hypoglycemic regimen presented to the emergency department with a three-day history of vomiting. Clinical examination revealed that the patient was febrile, hypotensive, tachypneic, and tachycardic. In addition, an abdominal examination revealed soft, lax abdominal tissue with acute discomfort in the left flank.

Laboratory testing showed that her creatinine level was 1.8 mg/dL, high random blood glucose level of 600 mg/dL, leukocytosis of 11.9 × 109/L, and hyponatremia of 125 mmol/L. Urinalysis showed leukocyte esterase positivity and pyuria (25-50 p/HPF), and urine culture had no growth. Computed tomography (CT) of the abdomen and pelvis with contrast revealed basal atelectasis and left pleural effusion, as well as gas in the left kidney parenchyma, which is highly suggestive of EPN associated with the adjacent excretory system, indicating pyelitis. Perinephric fat stranding and minor pararenal fluid were also observed.

**Figure 1:**
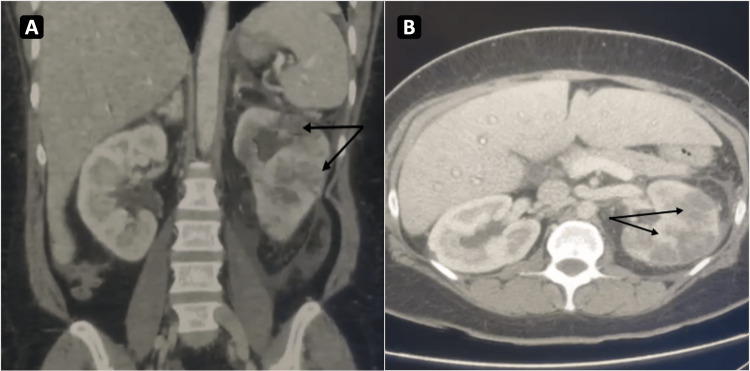
CT scan before intervention (A) Coronal sectional view of enhanced CT of the abdomen with contrast revealed a normal right kidney and a mildly enlarged left kidney with heterogeneous enhancement (black arrows) showing multiple wedge-like hypodense areas involving the renal cortex. (B) Cross-sectional view of the enhanced CT of the abdomen revealed signs of emphysematous changes (black arrows) and air pockets communicating with the adjacent pelvicalyceal system.

The patient was promptly admitted to the intensive care unit (ICU) owing to septic shock secondary to EPN with diabetic ketoacidosis. She was treated with intravenous fluids, inotropic support, wide-spectrum antibiotics (Tazocin), and glycemic management, and her clinical performance was monitored. Her blood culture was positive for *Klebsiella pneumoniae*, but her urine culture was negative. Her antibiotic regimen was subsequently modified in accordance with culture sensitivity. A COVID-19 swab test was performed during her stay in the ICU, and a positive result was obtained. Chest radiography revealed bilateral lung infiltrations. In addition, repeated CT scans showed that the left kidney's EPN was progressing, with the development of two collections with air-fluid: one at the upper pole (4.7×5.1×3.9 cm) and the other mid-kidney with two communicating components (5.5×6.6×8 cm; Figure 1). An intervention radiologist performed left percutaneous drainage and removed 100 mL of pus. The pus was sent for culture, and the results revealed the presence of *Klebsiella pneumoniae*. A repeat CT scan revealed that the drain tip had been positioned away from the collections. As such, the drain tip was repositioned, and the draining was restarted.

**Figure 2:**
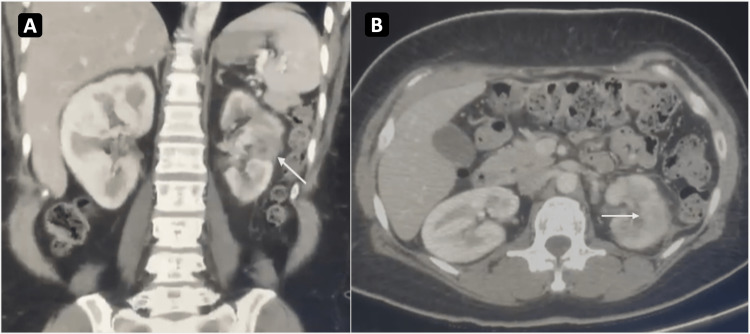
CT scan 2.5 months after discharge (A) Coronal section of enhanced CT of the abdomen with contrast revealed a left kidney interval decrease in size (white arrow) and resolution of previous hypodense lesions. (B) Cross-sectional view of enhanced CT of the abdomen revealed improved signs of emphysematous changes (white arrow). There was no air within the small residual collection.

On the 30th day of admission, the patient was discharged with two weeks of intravenous ertapenem. After the completion of the antibiotic course, the drain was observed for two days and showed no output. A week after that, an ultrasound scan was done and revealed no additional collection. The patient presented for the follow-up to the clinic 2.5 months after being discharged and was noted to be asymptomatic. Repeated laboratory testing revealed that her renal function was normal. A repeat CT scan also revealed the complete disappearance of the collections (Figure 2).

Currently, the patient is in good clinical health, with normal radiological findings during her most recent follow-up in our clinic.

## Discussion

EPN is a rare, life-threatening urological emergency with severe necrotizing infection characterized by gas formation in the renal parenchyma and collecting system as a result of gas-producing organisms. Patients with severe sepsis are more likely to develop EPN [1,2]. Risk factors for EPN include intravenous drug use, alcoholism, malnutrition, obstructive uropathy, or anatomical abnormalities in the urinary system. EPN is also frequently observed in individuals who are immunocompromised or with diabetes. Diabetes is a key factor in the development of EPN [2,3].

*Escherichia coli* is the most prevalent organism found in urine cultures of patients with EPN. *Klebsiella* is the second most common organism. Other less common associated organisms include *Aerobacter aerogenes, Aspergillus fumigatus, Bactericides, Candida albicans, Clostridium septicum, Cryptococcus neoformans, Entameba histolytica, Proteus mirabilis, Proteus species*, and *Pseudomonas aeruginosa. Klebsiella* and *Proteus* were identified in one of ten percent of EPN cases with polymicrobial infections [3,4]. High tissue glucose levels, poor vascular blood flow, the presence of gas-forming microorganisms, obstruction of the urinary system, and weakened host defenses are all risk factors for the pathogenesis of EPN. Anaerobic metabolism is facilitated by high tissue glucose levels and decreased kidney blood flow, both of which are common in patients with diabetes [3,4].

EPN clinical presentations range from asymptomatic to severe sepsis. The condition may indicate that the patient has severe acute pyelonephritis. This indication is based on the presence of the following non-specific findings: pyrexia, fever, flank discomfort, and pyuria, which may impede the diagnosis and treatment of EPN. Additionally, the patient may experience abdominal discomfort, nausea, vomiting, dysuria, flank crepitations, pneumaturia, shock, renal angle tenderness, and a reduced level of consciousness [1-4]. Subcutaneous emphysema, pneumomediastinum, and multiple septic emboli in the brain, liver, and lungs are examples of rare presentations of EPN. Severe EPN may lead to multi-organ failure and septic shock, with a death rate of 20-80%. Altered mental status, acute renal failure, thrombocytopenia, and sepsis are poor prognostic factors during the diagnosis of EPN. Previous studies have shown that factors such as site infection, blood glucose level, sex, and age do not play a role in determining the prognosis [1-4].

Radiology is the primary imaging modality used in the diagnosis of EPN. The renal and perirenal areas of a plain kidney, ureter and bladder radiograph image can exhibit unusual gas shadows. In addition, ultrasound imaging may show a low-level acoustic shadow and high-amplitude echoes. However, an abdominal CT is the gold standard radiological technique to confirm the diagnosis of EPN. Abdominal CT scans reveal the degree of parenchymal damage and intrarenal gas and the extent of perirenal involvement [1-2]. Two EPN classification systems, based on CT findings, have been reported in the literature. The first classification was developed by Wan et al. [5]. This system was used to classify EPN cases into two types, each of which had a prognostic rule. Patients with renal necrosis and gas but no fluid collection were classified as type I. Type I patients have a mortality rate of 70%. Patients with parenchymal gas and fluid collection in the parenchyma, perinephric space, or collecting system were classified as type II. Type II patients have a mortality rate of 16% (Table 1).

**Table 1:** Wan et al. emphysematous pyelonephritis classification Source: [5]

Type	CT scan findings	Characterized by	Mortality rate
1	Renal necrosis with the presence of gas but no fluid	Reduced immune response limits the formation of pus collection and this leads to the spread of the inflammation culminating in a fulminant course of the disease	70%
2	Parenchymal gas associated with fluid in renal parenchyma, perinephric space, or collecting system	A better immune response results in the formation of pus in the kidney, leading to a slower course of the disease and a better prognosis	16%

Huang and Tseng similarly provided a thorough staging system [6]. The greater predictive value of this classification is used to select a management strategy. Huang and Tseng classified EPN cases into four categories on the basis of the gas's location and the disease's laterality (Table 2) [2]. These EPN cases are typically managed with empirical antimicrobial therapy followed by nephrectomies. Our case unequivocally shows that prompt antibiotic medication followed by percutaneous drainage enhances patient outcomes. This strategy is particularly important in managing comorbid patients in whom the surgical approach can be avoided [4, 7-14]. Patient risk factors, particularly poor glucose management and immune system disorders in patients with uncontrolled diabetes, significantly influence EPN. The clinical presentations of these patients may range from minor symptoms to severe sepsis. Therefore, clinical assessments are poor diagnostic tools for diagnosing EPN. Radiological imaging modalities are the standard diagnostic and prognostic tools used to diagnose and treat EPN [1,2,10-14].

**Table 2:** Huang and Tseng emphysematous pyelonephritis classification EPN - emphysematous pyelonephritis Source: [6]

Class	Sub-class	CT scan findings	Management plan
Class I		Gas in the collecting system only	Percutaneous procedures and antibiotics
Class II		Parenchymal gas only	
Class III	Class IIIA, Class IIIB	Extension of gas into perinephric space Extension of gas into pararenal space	Less than two risk factors: 85% survival rate with percutaneous drainage and antibiotics
Class IV		EPN in a solitary kidney or bilateral disease	Two or more risk factors: 92% failure rate with percutaneous drainage and antibiotics

Nephrectomy is the preferred method to manage EPN, despite being associated with a 40-50% morbidity. Previous studies indicate that the conservative management of EPN includes fluid resuscitation, rigorous glycemic control, antibiotic therapy, and the alleviation of urinary tract obstruction [1-4]. A multidisciplinary approach is required to ensure the best outcome for EPN patients. The multidisciplinary team should include urologists, nephrologists, and infectious disease specialists. Other management options, excluding surgery, include conservative management with long-term antibiotics, with or without percutaneous drainage [1,3,9,13].

Recently developed minimally invasive techniques reduce mortality and prevent renal failure onset. Numerous studies have shown that a conservative approach, appropriate metabolic control, and antibiotic administration are effective management strategies for EPN. We hypothesized that this conservative approach includes percutaneous drainage in the following cases: 1) bilateral EPN, 2) localized EPN, 3) EPN identified in a solitary kidney, and 4) EPN patients who are unfit for surgery due to the presence of significant comorbidities (as seen in our case). Therefore, a conservative approach is an effective treatment strategy for EPN, reducing the associated morbidities and mortalities. Numerous studies support this conservative approach [3,7,9].

A literature review of case reports on EPN revealed that the early diagnosis of EPN correlates with a decreased mortality rate. The presence of comorbidities has a profound impact on this correlation. A large cohort analysis showed that patients who received antibiotics and percutaneous drainage with an 18-month follow-up period had lower mortality rates. Radiological evidence of progressive renal necrosis, in the presence of biochemical indicators of escalating disease severity such as hypoalbuminemia, serves as a guide to indicate the failure of conservative management [1-3,14]. Further analysis of a comparable cohort showed that timely nephrectomies in patients with failed conservative care increase their survival and decrease long-term morbidities [2,10,11].

EPN is a severe necrotizing illness of the renal parenchyma. It has a high mortality rate and leads to a rapid onset of septic shock. In addition to pain, chronic sepsis, and thrombocytopenia, it has a variety of other presenting signs and symptoms which pose a challenge to its clinical diagnosis in patients. CT imaging provides an accurate method to diagnose and monitor EPN progression and treatment. Therefore, CT imaging with contrast is a great diagnostic utility in managing EPN [1-4,7-14]. Herein, we identified that, in the acute setting, early percutaneous drainage in conjunction with the initiation of appropriate antibiotics is a suitable therapeutic alternative to surgical nephrectomy in the management of EPN. Even so, as noted in the literature, prompt nephrectomies are associated with increased survival in patients who fail minimally invasive therapy.

## Conclusions

EPN is a rare, life-threatening disease. The conventional management of EPN includes an immediate nephrectomy, which has a significant mortality rate. However, the mortality rate of EPN is decreasing due to enhanced imaging techniques, effective antibiotics, and percutaneous catheter drainage. We recommend that all surgeons report cases of EPN inclusive of their management plans to aid in a substantial meta-analysis study. This meta-analysis study will contribute to developing precise guidelines for the management of EPN. In addition, the prognostic factors for EPN should be identified to classify patients. The classification of patients based on their prognostic factors will aid in the decision of whether to proceed with conservative or surgical management of the patient.
